# Pediatric Ingestion of Multiple Button Batteries

**DOI:** 10.5811/cpcem.2018.11.39538

**Published:** 2019-02-20

**Authors:** Johnny Fong, Tony Zitek

**Affiliations:** * University of Nevada, Las Vegas School of Medicine, University Medical Center of Southern Nevada, Department of Emergency Medicine, Las Vegas, Nevada; † Kendall Regional Medical Center, Department of Emergency Medicine, Miami, Florida

## CASE PRESENTATION

**Image 1:**
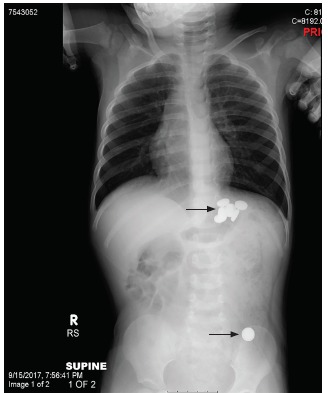
Supine radiograph showing multiple button batteries (arrows) approximately four hours after ingestion.

A two-year-old male presented to the pediatric emergency department for possible foreign body ingestion. Two hours prior to arrival, the child was found with the packaging for 10 button batteries, but his mother was only able to find one battery. The patient had no symptoms. Physical exam was within normal limits. Radiographs (Image 1) showed six foreign bodies within the stomach and one distally.

## DISCUSSION

Button battery ingestions are increasing in both frequency as well as in major or fatal outcomes.^1^ A low threshold for imaging is important as 54% of fatalities are from misdiagnosis due to non-specific presentations.^1^ Possible complications include perforations, fistula, strictures, hemorrhage, and death.^2^ The National Capital Poison Center Button Battery Ingestion Triage and Treatment Guideline (National Button Battery Guideline) specifically addresses patient age, battery size and location, and symptoms; however; it does not specifically address ingestion of multiple button batteries.^3^

The National Button Battery Guideline was recently changed to include the immediate administration of oral honey. This update was based on a recent study showing both in vitro and in vivo protective effects of honey in button battery ingestion.^4^ Imaging is not required if a specific set of criteria are met; otherwise, radiographs should be obtained of the entire length of the gastrointestinal tract to locate the battery.^3^ The main considerations are whether the battery is in the esophagus, if a magnet was co-ingested, or if the patient is having any symptoms.^3^ In these cases, endoscopic removal is preferred; however, surgical removal may be necessary if the battery is beyond reach.^3^ Important consideration should also be given to delayed injuries after battery removal.^3^

**CPC-EM Capsule**

What do we already know about this clinical entity?

*The National Button Battery Guideline does not offer specific recommendations for multiple battery ingestions, an entity which is not well discussed in the existing literature*.

What is the major impact of the image(s)?

*Through providing initial and subsequent radiographs, this case aims to increase awareness of button battery ingestions, which is increasing in both frequency and major outcomes*.

How might this improve emergency medicine practice?

*This case offers a successful example of diagnosis and management of a multiple button battery ingestion through admission for whole-bowel irrigation with polyethylene glycol*.

**Image 2:**
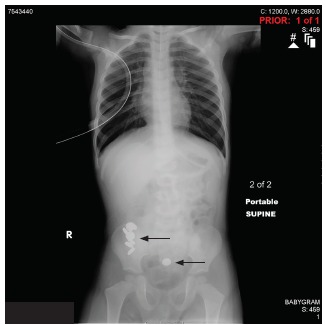
Supine radiograph showing progression of multiple button batteries (arrows) after 14 hours of whole-bowel irrigation.

This patient was admitted for observation, serial abdominal exams, and polyethylene glycol whole-bowel irrigation. Radiograph the next morning showed progression of the batteries (Image 2). Whole-bowel irrigation continued and eventually nine button batteries were passed rectally. Follow-up radiographs did not show any retained batteries.
